# A Unique Case of Unilateral Vocal Cord Palsy Following an Electrocution Injury

**DOI:** 10.7759/cureus.45443

**Published:** 2023-09-18

**Authors:** Akash Varshney, Ankita Semwal, Akhilesh Chandra Yadav, Kajal Mahto, Deepak Sangwan, Shriya Bhattarai, Amit Kumar Tyagi

**Affiliations:** 1 Department of Otorhinolaryngology-Head and Neck Surgery, All India Institute of Medical Sciences, Rishikesh, IND

**Keywords:** vagus, phonatory gap, recurrent laryngeal nerve, electric injury, vocal cord palsy

## Abstract

Electric injuries (in the form of lightning or electric shock) may lead to various implications in the human body, the most important of which include neurological insults. The damage caused is influenced by the route of its entry into the body, its strength, and the duration of exposure. The muscles of the larynx receive motor supply from the recurrent laryngeal nerve (RLN) (except cricothyroid, which gets innervation from the external laryngeal nerve). Recurrent laryngeal nerve (RLN) palsy leading to vocal cord palsy is seen in several pathologies, but after thorough research of existing literature, we could only find a single case of vocal cord palsy following electric injuries, which was also lost in follow-up. In this report, we present a case of unilateral vocal cord palsy following an electric injury on the ipsilateral arm of a young male. He presented to the emergency department of our center soon after the accident. A multidisciplinary team was engaged in the overall management of the patient (in view of pleural effusion, acute kidney injury, and burn injury). He was started on steroids, speech therapy, and other supportive management. On follow-up, his condition improved, and laryngeal endoscopy showed positive signs. This case highlights a unique but rare possibility of vocal cord palsy following electric injuries and may help in the prompt diagnosis and management of the same.

## Introduction

Electric current, when passing through the body, may interfere with the normal functioning of the internal organs and may burn susceptible tissues. The severity of the injury is influenced by the pathway through which the current enters the body, the duration of its exposure, and the resistance faced by tissues [1]. Such electrocution accidents are not uncommon in the modern world. Over a thousand people die every year of electric injuries in the United States of America (USA), with a bimodal age distribution, with the first peak being less than six years of age and the other one being young adults [2]. India reported 9,059 and 8,945 deaths from electric injuries in the years 2010 and 2011 [3]. Tissues such as the skin, fat, and bones, with their high resistance to electric current, tend to heat up and coagulate on exposure to electric current. On the other hand, blood vessels and nerves, due to their low resistance, conduct the electric current [2]. Similarly, such accidents may also lead to perplexing neurological and psychological sequelae, which include depression, post-traumatic stress disorder, amyotrophic lateral sclerosis, parkinsonism, personality changes, and cognitive impairment [4].

## Case presentation

We hereby present a unique case report from an accident in hilly and remote areas of Uttarakhand, India, where a high-voltage wire (allegedly of 1,000 volts) fell on an under-construction metallic bridge. Several people were struck, and a few lost their lives or got severely injured. A 35-year-old, previously healthy male also got trapped on the bridge, and his left arm got scorched by the electric current. After the accident, he fell unconscious and was brought to the emergency room of our institute, where he was initially resuscitated and treated for burn injuries. He acquired a third-degree asymmetrical burn on the lateral aspect of his left arm, accounting for approximately 2% of his body surface area. He subsequently developed acute kidney injury, which was probably the consequence of degraded blood products in his urine. He developed mild difficulty breathing, which was evaluated, and he was found to have bilateral pleural effusion along with ascites. The nephrology, neurosurgery, and burns and plastic surgery teams were involved in the multidisciplinary management of the patient. Meanwhile, he started complaining of a mild sore throat and a change in his voice (hoarseness) the next day. He was thoroughly examined, and on laryngeal endoscopy (Video 1), his left true vocal cord was found to be paralyzed. High-resolution tomography (HRCT) was done, which did not show any organic cause causing damage to the recurrent laryngeal nerve (RLN). Other cranial nerves were examined and found to be normal. The point to be mentioned here is that he did not undergo intubation/bronchoscopy. After ruling out all possible contraindications, he was started on an injection dexamethasone 8 mg intravenous thrice a day for five days, which was tapered gradually. The patient was advised for speech therapy. He was followed up in the OPD after two and four weeks. His symptoms in terms of speech improved. On repeat laryngeal endoscopy (Video 2), some movement was noted in the left vocal cord, and phonatory gap reduced. The patient would be further followed up and evaluated.

**Video 1:** Laryngeal endoscopy at the time of presentation

**Video 2:** Laryngeal endoscopy at the time of follow-up

Table 1 presents the patient’s blood investigation during admission and discharge.

**Table 1:** Blood investigations of the patient at the time of admission and at the time of discharge SGOT: serum glutamic oxaloacetic transaminase, SGPT: serum glutamate pyruvate transaminase

Blood investigations	Values (at the time of admission)	Values (at the time of discharge)
Hemoglobin	13.8	12.5
Total leukocyte count	15,410	6,490
Urea/creatinine	99/6.25	76/0.76
SGOT/SGPT	73/91	23/116

## Discussion

The recurrent laryngeal nerve (RLN), which supplies all the muscles of the larynx (except the cricothyroid), is a branch of the 10th cranial nerve, the vagus. On the right side, it arcs around the right subclavian artery, and on the left, around the aorta, making it more susceptible to injury owing to its long course. Insults to the vagus or RLN during its course can lead to vocal cord paralysis. Patients with a single-sided immobile vocal cord may be asymptomatic in as many as 30%-50% of cases. The most frequent causes of RLN palsy are tumors, surgeries, idiopathy, and post-intubation [5].

Injuries due to electric current can be explained in three different ways. The first probable mechanism is the tissue destruction and coagulative necrosis caused by the conversion of electric energy into thermal energy. The second mechanism proposed is the alteration of cell membrane potential and tetanization caused by direct damage due to current. The last is the indirect injuries by trauma as a result of falls of surrounding objects/violent contraction of muscles [1].

Neurological deficits are not uncommon following electrocution and manifest in different forms. After extensive research of previously available literature, only a single case of bilateral vocal cord palsy was found. The unique case of unilateral vocal cord palsy after electric shock injury thus opens the gateway for unexplored, but further intertwined loops in the medical sciences.

The patient reported here sustained an electric injury that entered his body through his left upper arm, and the electric current, in a very possible way, traveled in a retrograde fashion to his upper torso, including the head and neck region. As the nervous system has low resistance to the flow of external electric current, it is more susceptible to electrothermal injuries. This exposure to an unnatural and aberrant electric current leads to hypoxic and vascular compromise in the neurons, which leads to cell death. Another postulated mechanism of nerve injury is the demyelinating injury caused by free radical injury, which leads to delayed impairment [6].

For further investigations, laryngeal electromyography (LEMG) could be deployed to distinguish the cause of palsy to be hidden in upper/lower motor neurons, peripheral nerves, neuromuscular junctions, muscle fibers, cartilages, or joints [6], although this could not be done in our case due to technical issues.

## Conclusions

Any case of hoarseness of voice or sore throat in a case of electrocution needs to be evaluated with a detailed history and laryngeal examination. After ruling out all possible anatomical/organic causes for recurrent laryngeal nerve palsy, a course of systemic steroids can be started along with speech therapy and other supportive management. Surgical intervention (thyroplasty) may be advocated in cases that do not respond adequately to medical management. Unilateral vocal cord palsy following electrocution may add a unique entity to the existing literature and needs to be further explored with the help of technological advancements.
